# A case of EDTA-dependent pseudothrombocytopenia: simple recognition of an underdiagnosed and misleading phenomenon

**DOI:** 10.1186/1472-6890-14-19

**Published:** 2014-05-01

**Authors:** Michael Nagler, Peter Keller, Daniel Siegrist, Lorenzo Alberio

**Affiliations:** 1 Department of Hematology and Central Hematology Laboratory, Inselspital University Hospital and University of Berne, CH-3010 Berne, Switzerland

**Keywords:** Thrombocytopenia, Laboratory hematology, Hematology analyzers

## Abstract

**Background:**

EDTA-dependent pseudothrombocytopenia (EDTA-PTCP) is a common laboratory phenomenon with a prevalence ranging from 0.1-2% in hospitalized patients to 15-17% in outpatients evaluated for isolated thrombocytopenia. Despite its harmlessness, EDTA-PTCP frequently leads to time-consuming, costly and even invasive diagnostic investigations. EDTA-PTCP is often overlooked because blood smears are not evaluated visually in routine practice and histograms as well as warning flags of hematology analyzers are not interpreted correctly. Nonetheless, EDTA-PTCP may be diagnosed easily even by general practitioners without any experiences in blood film examinations. This is the first report illustrating the typical patterns of a platelet (PLT) and white blood cell (WBC) histograms of hematology analyzers.

**Case presentation:**

A 37-year-old female patient of Caucasian origin was referred with suspected acute leukemia and the crew of the emergency unit arranged extensive investigations for work-up. However, examination of EDTA blood sample revealed atypical lymphocytes and an isolated thrombocytopenia together with typical patterns of WBC and PLT histograms: a serrated curve of the platelet histogram and a peculiar peak on the left side of the WBC histogram. EDTA-PTCP was confirmed by a normal platelet count when examining citrated blood.

**Conclusion:**

Awareness of typical PLT and WBC patterns may alert to the presence of EDTA-PTCP in routine laboratory practice helping to avoid unnecessary investigations and over-treatment.

## Background

**Figure 1:**
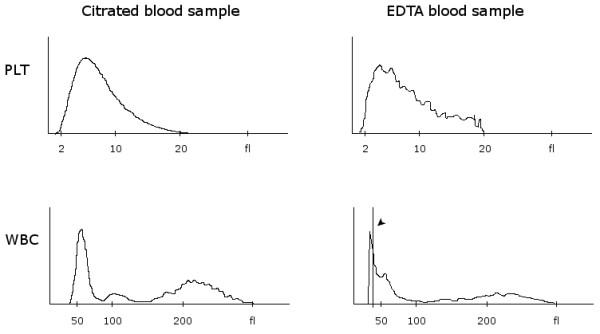
**Typical histograms of pseudothrombocytopenia in an EDTA sample (right) in contrast to normal histograms of a citrated (CPT) sample of the same patient (left).** Aggregated platelets are plotted as a serrated (“saw-teeth”) curve in the PLT histogram. In the WBC histogram, the largest aggregates are displayed as a peculiar peak on the left side (arrowhead).

EDTA-dependent pseudothrombocytopenia (EDTA-PTCP) is a common laboratory phenomenon. Its prevalence is reported to vary between 0.1-2% among hospitalized patients [1-3] and 15-17% in outpatients evaluated for isolated thrombocytopenia [4,5]. In contrast to serious and potential life-threatening causes of thrombocytopenia [6], EDTA-PTCP is solely an *in vitro* effect without any clinical relevance [7]. Cation chelation by EDTA leads to a conformational change of the platelet membrane GPIIb-IIIa complex unmasking a cryptic epitope, that becomes accessible for autoantibodies [8]. Antibodies are predominantly of IgG type but act as cold agglutinins that react with platelets *in vitro*. Hematology analyzers count the resulting platelet clumps as single giant platelets or as small lymphocytes in the white blood cell gate and indicate thrombocytopenia. Despite its harmlessness, EDTA-PTCP may generate significant costs and discomfort to the patient due to needless diagnostic testing, unnecessary transfusions and even withhold of emergency treatments [8-12]. Often, EDTA-PTCP remains unnoticed because blood smears are not routinely evaluated by visual inspection and warning flags as well as histograms of hematology analyzers are not interpreted correctly. However, EDTA-PTCP may be diagnosed easily even by general practitioners without any experiences in blood film examinations as aggregated platelets lead to typical changes of platelet (Figure 1; PLT) and white blood cell histograms (Figure 1; WBC) [1]. To the best of our knowledge, this typical pattern of the platelet histogram has not been published so far.

Visual evaluation of blood smears is regarded as gold standard for detection of EDTA-PTCP, but only a limited amount of smears will be performed in routine laboratories. A simpler approach for detection of EDTA-PTCP is to inspect the histograms and flags of hematology analyzers. Although proper diagnostic accuracy studies have not been done and previous investigations using former models of hematology analyzer suggest some false-positive and false-negative results [1,13], EDTA-PTCP is expected to be diagnosed correctly in most cases by this approach [8,14]. In our practice, we visually evaluate blood smears in all cases with new or unexpected thrombocytopenia below 70 × 10^6^/μl, and in cases with the typical histogram patterns or the respective flags of the hematology analyzer.

Which strategies can be then applied to determine the correct platelet count in daily practice? Several alternative anticoagulants have been investigated, but most of them are either not applicable to current hematology analyzers, or may induce pseudothrombocytopenia by themselves [8]. In fact, besides EDTA, pseudothrombocytopenia was also recognised in samples anticoagulated with oxalate, heparin, and hirudin and even citrate [15,16]. This *in vitro* phenomenon was not observed in samples anticoagulated with mixtures of EDTA and aminoglycosides [17,18], with magnesium salt [19] and with the CPT mixture (citrate 17 mmol/l, pyridoxal 5′-phosphate 11.3 mmol/l and Tris 24.76 mmol/l) [8,18,20]. It is reported, that immediate processing of the blood samples and collection of the samples in pre-warmed tubes reduces the presence of platelet aggregates [7]. However, this manoeuvre will be possible in special settings only. In our laboratory if platelet aggregates are found, we confirm EDTA-PTCP and assess the correct platelet count by obtaining a new sample using CPT as anticoagulant.

## Case presentation

**Figure 2:**
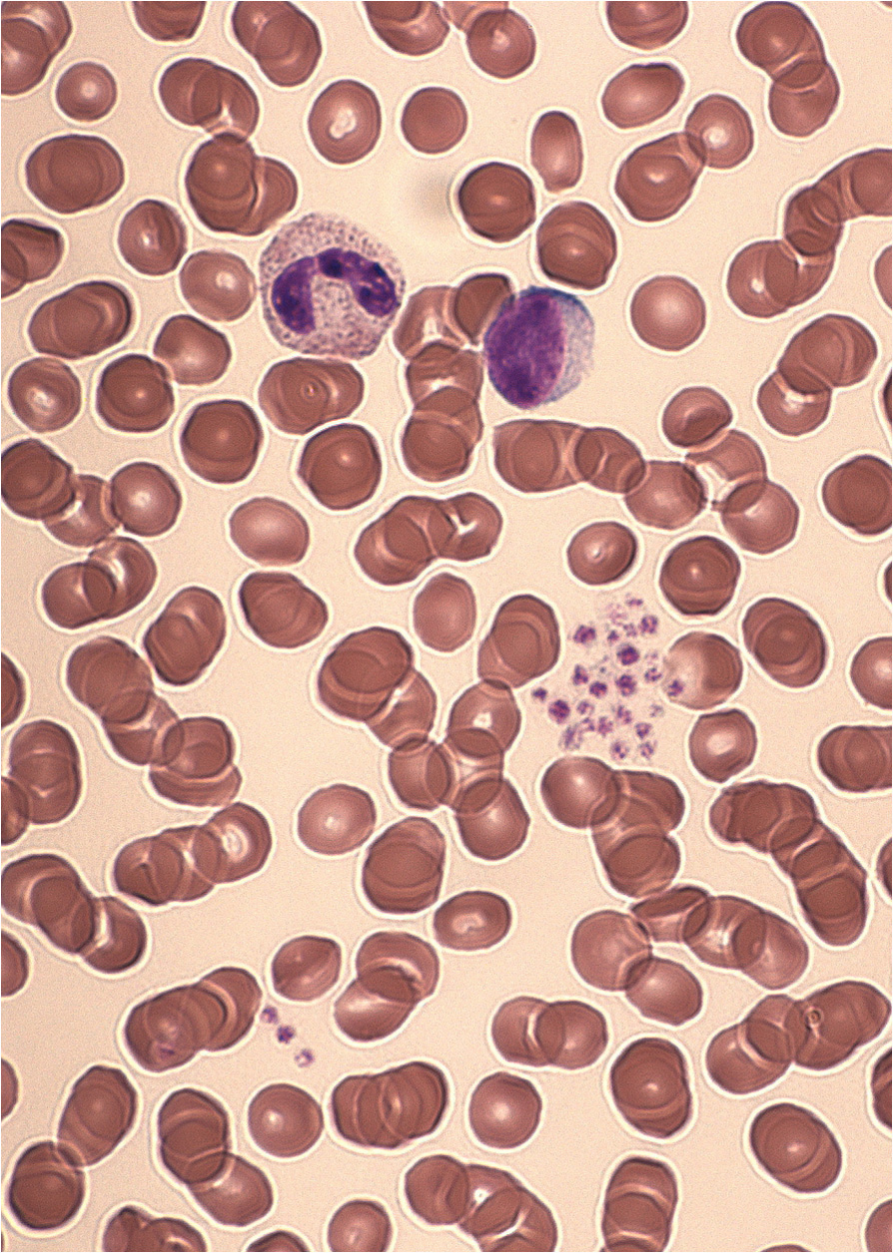
**Blood smear of an EDTA sample showing activated lymphocytes and platelet aggregates.** Patient was referred with suspected acute leukemia because lymphocytes were misinterpreted as blasts and thrombocytopenia was not recognised as EDTA-dependent pseudothrombocytopenia.

A 37-year-old female patient of caucasian origin was referred from a regional hospital with suspected acute leukemia. The referring physician reported on fever, cough, severe thrombocytopenia and irregular cells in the blood smear. Emergency unit crew arranged extensive laboratory investigations, ordered a CT scan, asked for bone marrow biopsy, and reserved a platelet concentrate. Examination of EDTA blood by an automated hematology analyzer (Coulter Counter LH750, Beckman-Coulter Inc., Nyon, Switzerland) showed an isolated thrombocytopenia (38 × 10^6^/μl) as well as typical patterns of platelet and WBC histograms. The aggregated platelets lead to an serrated (“saw-teeth”) curve of the platelet histogram (Figure 1) and the largest platelet aggregates are plotted as a peculiar peak on the left side of the WBC histogram (Figure 1; arrowhead). Furthermore, hematology analyzer reported on the following flags: “platelet clumps” and “giant platelets”. Visual inspection of the blood smear revealed activated lymphocytes and platelet aggregates (Figure 2). EDTA-dependent pseudothrombocytopenia (EDTA-PTCP) was confirmed by a normal platelet count when examining CPT-anticoagulated blood (173 × 106/μl). Due to normalisation of PLT, no blood smear of the citrated sample was performed. Review of previous laboratory tests with the family physician revealed normal PLT values. Thus, activated lymphocytes as well as EDTA-PTCP were interpreted as secondary to upper airway infection [8,21].

## Conclusions

In conclusion, this case illustrates typical patterns of platelet and WBC histograms on automated hematology analyzers in EDTA-PTCP (Figure 1). Awareness of these patterns may alert to the presence of EDTA-PTCP in routine clinical practice. This may help physicians as well as laboratory personnel to be aware of EDTA-PTCP and to prevent unnecessary investigations as well as over-treatment.

## Consent

Written informed consent was obtained from the patient for publication of this Case report and any accompanying images. A copy of the written consent is available for review by the Editor of this journal.

## Abbreviations

EDTA-PTCP: EDTA-dependent pseudothrombocytopenia; WBC: White blood cell.

## Competing interests

The authors declare that they have no competing interests.

## Authors’ contributions

MN and DS looked after the patient, conducted the blood smear and interpreted the histogram of the hematology analyzer. PK and LA supervised hematologists on duty. MN wrote the first draw of the manuscript. All authors revised the manuscript. All authors read and approved the final manuscript.

## Pre-publication history

The pre-publication history for this paper can be accessed here:

http://www.biomedcentral.com/1472-6890/14/19/prepub
